# Comparison of total, salivary and calculated free cortisol levels in patients with severe sepsis

**DOI:** 10.1186/s40560-015-0125-0

**Published:** 2016-01-08

**Authors:** Gulsah Elbuken, Zuleyha Karaca, Fatih Tanriverdi, Kursad Unluhizarci, Murat Sungur, Mehmet Doganay, Fahrettin Kelestimur

**Affiliations:** Department of Endocrinology and Metabolism, Erciyes University Medical School, 38039 Kayseri, Turkey; Department of Internal Medicine, Erciyes University Medical School, Kayseri, Turkey; Department of Infectious Diseases, Erciyes University Medical School, Kayseri, Turkey

**Keywords:** ACTH stimulation test, HPA axis, Total cortisol, Salivary cortisol, Calculated free cortisol, Severe sepsis

## Abstract

**Background:**

The purposes of the study were to compare serum total cortisol (STC), salivary cortisol (SaC) and calculated free cortisol (cFC) levels at baseline and after the adrenocorticotrophic hormone (ACTH) stimulation test in patients with severe sepsis (SS) and determine the suitability of use of SaC and cFC levels instead of STC for the diagnosis of adrenal insufficiency (AI) in patients with SS. And secondary aims of this study were to compare these parameters in patients with SS with healthy controls and check their effects on survival status of the patients.

**Methods:**

Thirty patients with SS (15 men and 15 women) were compared with 16 healthy controls. Low-dose (1 μg) ACTH stimulation test was performed to the patients on the first, seventh and 28th days of diagnosis of SS, but in control group, 1 μg ACTH stimulation test was performed only once. STC, SaC and cFC levels were measured during ACTH stimulation test.

**Results:**

Patients were categorized as having low or high baseline STC according to a cut-off level of 10 μg/dL. In high STC group, baseline and peak SaC levels were found to be 2.3 (0.2–9.0) and 3.4 (0.5–17.8) μg/dL on D1 and 1.1 (0.8–4.6) and 2.6 (1.3–2.9) μg/dL on D7, respectively. In the control group, baseline and peak SaC levels were 0.4 (0.1–1.4) and 1.1 (0.4–2.5) μg/dL, respectively. Baseline and peak SaC levels after ACTH stimulation were found to be higher in high STC group than in controls, but they were found to be similar in low STC and control groups. In high STC group, cFC levels were 0.3 (0.1–0.3) and 0.4 (0.3–0.7) μg/dL on D1 and 0.2 (0.1–0.3) and 0.4 (0.1–0.7) μg/dL on D7, respectively. In the control group, baseline and peak cFC levels were 1.7 (0.4–1.9) and 1.8 (1.0–6.6) μg/dL, respectively. cFC levels were found to be lower in patients with SS subgroups than in the control group. Baseline and stimulated STC, SaC and cFC levels did not differ according to the survival status. SaC, cFC and STC levels were found to be correlated with each other.

**Conclusions:**

SS is associated with increased SaC, but decreased cFC levels when baseline STC is assumed to be sufficient. When STC level is assumed to be insufficient, SaC levels remain unchanged, but cFC levels are decreased. Lower STC levels is not associated with increased mortality in patients with SS. More data are needed in order to suggest the use of SaC and cFC instead of STC.

**Trial registration:**

ClinicalTrials.gov No: NCT02589431

## Background

In stress-free people, cortisol is secreted in diurnal pattern. Any type of acute illness or trauma results in loss of the diurnal variation of cortisol secretion [1]. In case of a critical illness, organism needs to maintain adequate cortisol levels to cope with the illness. Cortisol response to such an illness is one of the factors that determines survival [2]. Lower or higher levels of serum total cortisol (STC) levels than expected were reported to be closely related to survival status [3]. As a kind of critical illness, sepsis may lead to major changes in hypothalamo-pituitary-adrenal (HPA) axis that causes challenges in the evaluation of its functions in those patients [4, 5].

Relative adrenal insufficiency (RAI), or critical illness-related corticosteroid insufficiency (CIRCI), is a form of adrenal insufficiency (AI) in critically ill patients whose cortisol levels are inadequate for the severe stress response they experience [6]. The diagnosis of CIRCI and the indications for corticosteroid therapy in critical illness (CI) are still controversial [3, 6–10]. Baseline or random STC levels, cortisol increment after adrenocorticotrophic hormone (ACTH) stimulation test (delta cortisol), free cortisol (FC) levels, or other markers presenting FC, or a combination of these tests have been proposed as diagnostic tests for CIRCI [4, 8–11].

Annane et al. have reported a delta cortisol of less than 9 μg/dL or a random cortisol of less than 10 μg/dL as the best predictors of AI in patients with severe sepsis (SS) and septic shock [9]. But, in critically ill patients, since the adrenal glands might already be maximally stimulated, baseline cortisol levels may be higher than ACTH-stimulated serum cortisol levels [4, 5]. For this reason, some authors suggest measuring random serum cortisol levels at any time of the day regardless of cortisol diurnal rhythm [6, 9].

More than 90 % of circulating cortisol is predominantly bound to not only cortisol binding globulin (CBG) but also albumin [12]. Thus, in the presence of both hypoalbuminemia and decreased CBG levels, the ratio of bound to free cortisol levels can be altered. In this situation, measurement of FC becomes more important. Since direct FC measurement is time consuming and non-automated, some indirect methods to determine FC levels had been introduced such as Coolens’ method [12, 13]. In patients with CI, the synthesis of CBG and albumin is reduced leading to overestimation of AI if we only use STC levels. Since some studies demonstrated that salivary cortisol (SaC) reflects free or unbound plasma cortisol levels, this method is used more often in clinical studies [14–19]. STC and SaC levels at baseline and after ACTH stimulation had been used in some studies in patients with CI [17, 18].

The aims of the present study were to compare STC, SaC and calculated free cortisol (cFC) levels at baseline and after the ACTH stimulation test in patients with SS and determine the suitability of SaC and cFC levels instead of STC for the diagnosis of AI in patients with SS. And secondary aims of this study were to compare these parameters in patients with SS with healthy controls and check their effects on survival status of the patients.

## Patients and methods

The study was registered to Clinical Trials. Clinical trial Gov No: NCT02589431.

### Patients

Patients meeting the American College of Chest Physicians (ACCP)/Society of Critical Care Medicine (SCCM) consensus definition of SS [20] were enrolled from the Internal Medicine Intensive Care Unit (ICU) of our hospital. The study was approved by the Local Ethics Committee. Informed consent was obtained from all volunteers or their first-degree relatives. Exclusion criteria were the presence of malignancy, history of corticosteroid exposure, and also presence of any disease that could affect HPA axis. We also excluded the patients who have any visible bleeding in their oral cavity.

Thirthy patients with SS (15 men and 15 women) and 16 (8 men and 8 women) healthy controls were included in the study. The first 24 h of diagnosis of SS was shown as day (D) 1. Baseline hormone tests were obtained especially in the morning between 08.00–09.00 a.m. within 24 h after the symptoms of SS were detected (D1). HPA axis was evaluated by baseline STC, SaC and cFC levels, and STC, SaC and cFC response to low-dose (1 μg) ACTH stimulation test. ACTH stimulation test was also performed in the morning. The patients who were alive and accepted to continue in the participation of the study were evaluated again on seventh (D7) and 28th (D28) days of SS.

While the patients were treated for SS by his/her own physician in ICU, they were evaluated for the presence/absence of (relative) AI concurrently. AI was suspected/considered clinically in the presence of hemodynamic instability and/or hyponatremia-hyperkalemia. Patients, who were determined as AI by their treating physician and administered glucocorticoid therapy, were excluded from the study.

Apart from routine daily evaluations, blood samples were obtained from all the patients in accordance with the study protocol. All the blood and saliva samples were stored at −80 °C and assayed after the study protocol finished. Thus, although this is a prospective study, samples were analysed retrospectively.

**Fig. 1:**
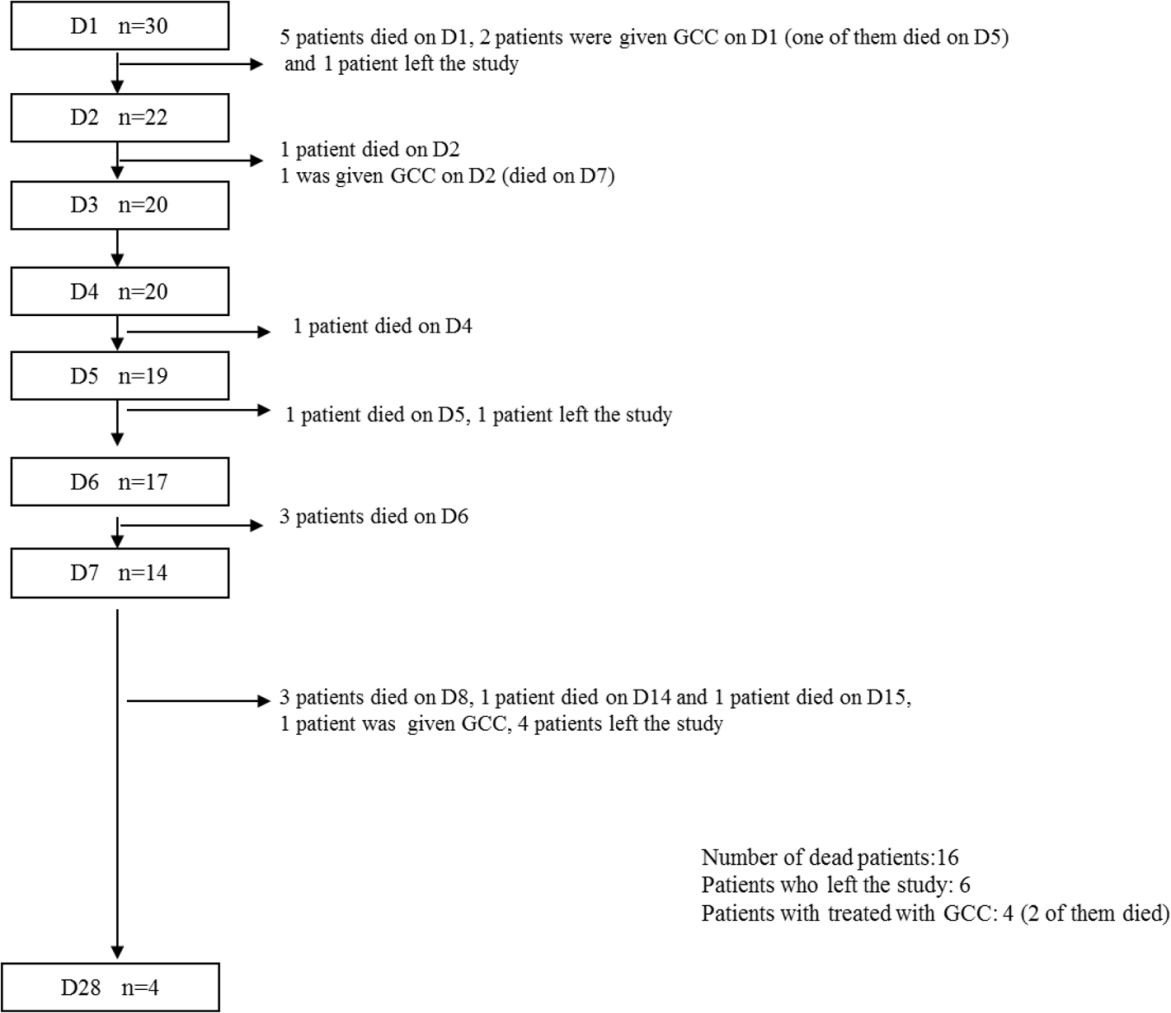
Patient enrollment

Low-dose ACTH stimulation test was performed on D1, D7 and D28 as described previously [21]. cFC levels were estimated by Coolens’ equation [13]. Blood and saliva samples were obtained before and 30 and 60 min after ACTH injection. STC, CBG and SaC levels were measured during the ACTH stimulation tests. We have used baseline and peak hormone levels after ACTH stimulation test to compare the groups. No adverse events were reported during the tests. Between D1–D7 of the study, only baseline STC, SaC and cFC levels were obtained. Acute Physiology and Chronic Health Evaluation (APACHE) II score [22] and Sequential Organ Failure Assessment (SOFA) score [23] were calculated for each patient on D1, D7 and D28. Data were compared with 16 healthy controls. Patient enrollment is summarized in Fig. 1.

Irrespective of the decision of the treating physician on the diagnosis of AI, we have categorized the patients retrospectively as low or high baseline STC group according to the baseline STC cut-off level of 10 μg/dL on D1, D7 and D28.

#### Assays

STC levels were measured by RIA method (Immunotech; Prag, Czech Republic) with an intraassay coefficient of variation 5.1 % and interassay coefficient of variation 9.2 %. Serum CBG concentrations were analysed with RIA (BioSource Europe S.A., Nivelles, Belgium). Intraassay and interassay coefficients of variation were 3.9 and 5.5 %, respectively. The Coolens' method was used to calculate serum FC concentration: FC^2^*K* (1 + *N*) + FC [1 + *N* + *K* (*G* − *T*)] − *T* = 0, where *K* = 3 × 10^−7^ M^−1^, M = affinity of CBG to cortisol at 37 °C, *G* = CBG, FC = free cortisol, *T* = cortisol and *N* = ratio of albumin bound to FC (1.74). FC was calculated as follows: $\mathrm{F}\mathrm{C}=\sqrt{Z^2+\frac{T}{\left(1+N\right)K}-Z}$, and $Z=\frac{1}{2K}+\frac{G-T}{2\left(1+N\right)}$ [13]. Saliva samples were collected, stored and analysed as described previously [24]. SaC was measured by using high-sensitivity enzyme immunassay (EI) kit (Salimetrics® Inc, State College, PA, USA), according to the manufacturer’s instructions [15, 17]. The interassay coefficient of variation over the range of low to high values varied from 5.7 to 6.8 %, whereas the respective intraassay coefficients of variation were 3.2 and 6.3 % [15].

### Statistical analysis

All statistical analysis were done by Statistical Package for Social Sciences (SPSS for Windows, version 15; Chicago; IL). Normal distribution of the data were tested by Shapiro-Wilk test. Since the data were not distributed normally, statistical analysis was done by nonparametric tests. The hormone results are presented as median, minimum and maximum levels. Statistical significance was set at *p* value less than 0.05.

## Results

There were no statistical difference between the mean ages of the patients of SS and the control group (61.7 ± 14.7 (range 23–82) and 58.7 ± 4.1 (range 51–64) years, respectively).

Among patients diagnosed with SS, the infection originated from the lung in 12 patients (40 %) (pneumonia), blood in 4 patients (13 %), urine in 8 patients (27 %), gastrointestinal tract in 4 patients (13 %) and skin and soft tissue in 2 patients (7 %). Mean leukocyte count of SS patients was 12,940 ± 7502/μL.

Comorbid conditions in these 30 SS patients included type 2 diabetes mellitus (DM) in 6 patients (20 %), coronary heart disease in 11 patients (37 %) and chronic obstructive pulmonary disease (COPD) in 4 patients (10 %). Nine patients (30 %) did not have any comorbidity. No remarkable comorbidity was present in control subjects.

Among 21 patients with comorbidities, 5 were using metformin, 2 were using metformin and gliclazide, 6 were using angiotensin converting enzyme inhibitors or angiotensin II receptor blockers and 1 was using an angiotensin II receptor blocker and a beta blocker combination therapy before development of SS. All of the patients with COPD were using short-acting beta-2 agonists, and none of them was using inhaled corticosteroids. Due to hypotension, the antihypertensive drugs were not administered to patients during SS. The patients with DM were followed with subcutaneous insulin treatment, and treatment regimens other than glucocorticoids inhaled treatments were administered to patients with COPD.

At the onset of SS, all 30 patients were found to have reduced blood pressure (100 %) and additionally hypothermia occurred in 5 patients (17 %), hyperkalemia in 2 patients (7 %) and hyponatremia in 4 patients (13 %). Growth of microorganisms was observed in body fluid cultures in 22 patients (%73) and 10 patients (33 %) required vasopressor infusions. A total of 4 patients (13 %) were administered glucocorticoid therapy according to the judgement of following physician. These 4 patients were excluded from further analysis in the study after steroid administration.

Patients were categorized as low or high STC group according to their baseline STC (low if STC <10 μg/dL, high if STC ≥10 μg/dL [9].

Thirty patients were included in the study on D1 and 11 patients were defined as low STC according to their baseline STC levels on D1. Low STC group had delta STC levels <9 μg/dL and peak STC levels <21 μg/dL on D1. Not only baseline STC but also peak STC, peak SaC and basal and peak cFC levels were also found to be significantly lower in patients with low STC group. Baseline and stimulated CBG levels on D1 were not different in two groups (Table 1).

**Table 1:** Baseline and peak CBG, cFC, SaC and STC levels on D1 and D7

Day of the study		D1			D7			Healthy	*p*1	*p*2	*p*3	*p*4	*p*5	*p*6	*p*7	*p*8
		All SS patients	SS with high STC	SS with low STC	All SS patients	SS with high STC	SS with low STC									
*n*		30	19	11	14	4	10	16								
STC (μg/dL)	Baseline	13.7 (3.1–53.2)	18.7 (10.4–53.2)	8.2 (3.1–9.4)	7.6 (0.4–13.9)	12.4 (12.0–13.9)	6.5 (0.4–9.6)	14.3 (4.3–20)	0.13	**<** *0.01*	**<** *0.01*	**<** *0.01*	**<** *0.01*	*0.01*	0.67	0.03
	Peak	20.3 (7.4–52.0)	31.0 (14.1–52.0)	11.0 (7.4–20.6)	12.7 (4.0–25.0)	18.9 (8.3–25.0)	12.4 (3.9–21.4)	27 (15.2–41.4)	0.64	*<0.01*	0.27	*<0.01*	*<0.01*	0.24	*0.02*	*<0.01*
SaC (μg/dL)	Baseline	1.9 (0.2–9.0)	2.3 (0.2–9.0)	0.9 (0.2–2.9)	0.7 (0.3–4.6)	1.1 (0.8–4.6)	0.6 (0.3–3.0)	0.4 (0.1–1.4)	*<0.01*	0.15	*<0.01*	0.09	0.04	0.07	0.06	0.72
	Peak	2.2 (0.5–17.8)	3.4 (0.5–17.8)	1.9 (0.6–4.2)	1.7 (0.2–4.0)	2.6 (1.3–2.9)	1.5 (0.2–4.0)	1.1 (0.4–2.5)	*<0.01*	*0.02*	*<0.01*	0.11	0.37	0.39	*0.01*	0.14
cFC (μg/dL)	Baseline	0.5 (0.1–1.9)	0.7 (0.4–1.9)	0.3 (0.1–0.3)	0.3 (0.1–0.5)	0.4 (0.4–0.5)	0.2 (0.1–0.3)	0.7 (0.4–1.9)	0.48	*<0.01*	*<0.01*	*<0.01*	*<0.01*	*0.01*	0.65	*<0.01*
	Peak	0.71 (0.3–1.9)	1.1 (0.5–1.9)	0.4 (0.3–0.7)	0.4 (0.1–0.9)	0.7 (0.3–0.9)	0.4 (0.1–0.7)	1.8 (1.0–6.6)	<*0.01*	<*0.01*	<*0.01*	<*0.01*	<*0.01*	0.24	0.89	0.12
CBG (μg/dL)	Baseline	0.3 (0.1–0.6)	0.3 (0.1–0.6)	0.2 (0.1–0.4)	0.3 (0.2–0.5)	0.4 (0.3–0.4)	0.3 (0.2–0.5)	0.5 (0.3–0.8)	**<** *0.01*	0.12	**<** *0.01*	**<** *0.01*	**<** *0.01*	0.94	**<** *0.01*	**<** *0.01*
	Peak	0.3 (0.1–0.5)	0.3 (0.1–0.5)	0.3 (0.1–0.5)	0.4 (0.1–0.5)	0.3 (0.2–0.4)	0.4 (0.1–0.5)	0.6 (0.4–0.9)	**<** *0.01*	0.6	**<** *0.01*	**<** *0.01*	**<** *0.01*	0.35	**<** *0.01*	**<** *0.01*

Abbreviations: *D1* first day of severe sepsis, *n* number of the subjects, *STC* serum total cortisol, *SaC* salivary cortisol, *cFC* calculated free cortisol, *CBG* cortisol binding globulin, *p1* belongs to comparison between hormon levels of the patients with severe sepsis on D1 and healthy control, *p2* belongs to comparison between patients who have basal STC ≥ or <10 μg/dL on D1, *p3* belongs to comparison between patients with severe sepsis who have baseline STC ≥10 μg/dL on D1 and healthy control, *p4* belongs to comparison between patients with severe sepsis who have baseline STC <10 μg/dL on D1 and healthy control, *p5* belongs to comparison between hormone levels of the patients with severe sepsis on D7 and healthy control, *p6* belongs to comparison between patients who have baseline STC ≥ or <10 μg/dL on D7, *p7* belongs to comparison between patients with sepsis who have baseline STC ≥10 μg/dL on D7 and healthy control, *p8* belongs to comparison between patients with sepsis who have baseline STC <10 μg/dL on D7 and healthy control

All of 5 patients died on D1 had high STC levels (41, 21, 25, 32, and 31 μg/dL, respectively). Five of 11 patients in low STC group died and 6 survived until D7. Among 6 patients who survived until D7, 1 patient had baseline STC levels 8.4 and 12.4 μg/dL on D1 and D7, respectively. The remaining 5 patients with low STC on D1 had also low STC on D7. Newly onset low STC levels were present in 5 patients on D7. Baseline cFC levels were also found to be lower in low STC group (Table 1). Baseline or peak SaC and cFC levels were not found to be different in low or high STC groups (Table 1).

No marked difference was observed between low or high STC groups in means of albumin values and APACHE II and SOFA scores. However, albumin levels were significantly lower compared to healthy group irrespective of the baseline STC (*p* < 0.01). Groups were compared at the end of D7 and D28 with respect to serum albumin levels obtained on D1 and D7 depending on survival. Serum albumin values did not differ between SS subgroups. Mean APACHE II score was 21 (min 7, max 37) on D1 and 12 (min 7, max 29) on D7 among survivors and 32 (min 16, max 40) on D1 and 33 (min 25, max 36) on D7 among non-survivors (*p* < 0.01). Mean SOFA score was 6 (min 1, max 12) on D1 and 4 (min 1, max 6) on D7 among survivors and 11 (min 3, max 21) on D1 and 13 (min 8, max 14) on D7 among non-survivors (*p* < 0.01).

Hormone levels between D1–D7 were compared in patients with SS who have survived or not until D7. On D5 and D7, baseline STC and cFC levels and, on D1, baseline STC and baseline and peak cFC levels were found to be lower in patients who have survived. There were no differences between SaC and CBG levels of the patients who have survived or not (Table 2). The patients with low baseline STC on D1 (*n* = 11) were divided into two groups according to their survival status until D7. Baseline and stimulated STC, SaC and cFC levels were not significantly different in patients who have survived or not. Similarly, if 19 patients with high baseline STC on D1 were divided into two groups according to their survival status until D7, baseline and stimulated STC, SaC and CFC levels were not found to be different in patients who have survived or not (data not shown).

**Table 2:** Differences between STC, SaC, cFC and CBG levels of the patients who have survived or not

Days	Number	Survival	STC (μg/dL)		SaC (μg/dL)		cFC (μg/dL)		CBG (μg/dL)	
			Baseline	Peak	Baseline	Peak	Baseline	Peak	Baseline	Peak
D1	30	Alive	9.4 (3.1–25.8)	14.3 (7.4–46.9)	1.0 (0.2–7.5)	2.0 (0.6–15.0)	0.3 (0.1–0.9)	0.5 (0.3–1.7)	0.3 (0.1–0.6)	0.4 (0.2–0.5)
		Dead	21.0 (4.6–53.2)	31.0 (7.5–52.0)	2.3 (0.2–9.0)	3.4 (0.5–17.8)	0.8 (0.2–1.87)	1.1 (0.3–1.9)	0.2 (0.1–0.5)	0.3 (0.1–0.5)
D2	22	Alive	12.4 (4.9–40.9)		0.7 (0.1–3.3)		0.4 (0.2–1.4)		0.2 (0.1–0.5)	
		Dead	17.7 (5.6–24.8)		0.6 (0.1–5.2)		0.8 (0.3–1.5)		0.2 (0.2–0.5)	
D3	20	Alive	10.2 (3.2–31.3)		0.7 (0.1–3.8)		0.3 (0.1–1.1)		0.2 (0.1–0.5)	
		Dead	22.8 (7.9–43.3)		0.9 (0.1–5.2)		0.8 (0.3–1.5)		0.2 (0.2–0.4)	
D4	20	Alive	12.6 (3.5–22.1)		0.8 (0.1–3.0)		0.5 (0.1–0.8)		0.3 (0.1–0.4)	
		Dead	18.6 (3.3–50.5)		2.2 (0.2–9.4)		0.7 (0.1–1.8)		0.3 (0.1–0.4)	
D5	19	Alive	11.4 (5.2–30.4)		0.8 (0.1–4.6)		0.4 (0.2–1.1)		0.3 (0.1–0.4)	
		Dead	20.2 (15.6–30.0)		0.7 (0.1–3.1)		0.7 (0.6–1.1)		0.2 (0.2–0.4)	
D6	17	Alive	10.4 (2.1–43.0)		1.0 (0.1–4.6)		0.4 (0.1–1.5)		0.2 (0.1–0.5)	
		Dead	27.3 (5.6–32.6)		2.0 (0.9–4)		1.0 (0.2–1.2)		0.3 (0.1–0.4)	
D7	14	Alive	6.9 (0.4–13.9)	9.9 (3.9–25.0)	0.5 (0.3–3.0)	1.7 (0.2–4.0)	0.2 (0.1–0.5)	0.4 (0.1–0.9)	0.3 (0.2–0.5)	0.4 (0.2–0.5)
		Dead	8.7 (5.6–12.4)	13.3 (8.3–18.8)	0.9 (0.6–4.6)	2.4 (0.6–2.9)	0.3 (0.2–0.4)	0.5 (0.3–0.7)	0.3 (0.2–0.5)	0.3 (0.1–0.4)
*p*1			*0.02*	0.53	0.28	0.12	*0.02*	*0.01*	0.39	0.27
*p*2			0.08		0.64		0.57		0.56	
*p*3			0.55		0.69		0.06		0.36	
*p*4			*0.02*		0.32		0.55		0.91	
*p*5			*0.02*		0.9		*0.02*		0.44	
*p*6			0.3		0.2		0.3		0.5	
*p*7			*0.01*	0.24	0.07	0.39	*0.01*	0.24	0.94	0.35

Abbreviations: *STC* serum total cortisol, *SaC* salivary cortisol, *cFC* calculated free cortisol, *CBG* cortisol binding globulin, *D* day of severe sepsis, *p1*, *p2*, *p3*, *p4*, *p5*, *p6* and *p7* belongs to comparisons between patients with severe sepsis on D1, D2, D3, D4, D5, D6 and D7 who have survived or not

In total, 11 patients died before D7, 3 patients were treated with glucocorticoid between D1 and D7 (and 2 of them died before D7); 5 patients died before D28, 1 was given glucocorticoid between D7 and D28. At the end of 28 days, 8 patients were alive.

Nineteen patients had high STC on D1,  3 of them were given glucocorticoids  (2 died), 6 of them died, 2 of them left the study and 8 were alive at the end of 7 days. Four patients had high STC on D7, 2 died, 1 were given glucocorticoid and 1 were alive at the end of 28 days.

Five of 11 patients with low STC on D1 died and 6 were alive at the end of 7 days. One of the patients with low STC group on D1 was recovered until D7. On D7, 5 new patients with baseline STC levels lower than 10 μg/dL were added to lower STC group (final number was 10). Three of these 10 patients with low STC value on D7 died and 7 of them were alive at the end of 28 days. One of the 8 patients who were alive on D28 was in high STC group. Although 8 patients were alive, only 4 patients could participate our study on D28 of SS. The individual data of the these 4 patients on D28 are summarized in Table 3.

**Table 3:** Individual data of 4 patients on D28

No	Age	Gender	STC (μg/dL)		SaC (μg/dL)		cFC (μg/dL)		CBG (μg/dL)		Albumin (mg/dL)	APACHE II score	SOFA score	Survival
			Baseline	Peak	Baseline	Peak	Baseline	Peak	Baseline	Peak				
5	76	M	0.4	3.8	0.4	1.0	0.1	0.1	0.2	0.3	1.9	20	6	Died on D57
6	60	F	9.6	12.9	1.7	1.7	0.3	0.4	0.4	0.5	3.5	16	4	Alive
8	81	F	18.9	51.2	0.7	1.3	0.4	0.9	0.3	0.4	3.2	16	4	Alive
13	45	M	30.9	25.2	1.2	2.2	1.1	0.9	0.2	0.4	2.7	6	3	Alive

Abbreviations: *STC* serum total cortisol, *SaC* salivary cortisol, *cFC* calculated free cortisol, *CBG* cortisol binding globulin, *D* day of severe sepsis, *M* male, *F* female, *APACHE II* Acute Physiology and Chronic Health Evaluation II, *SOFA* Sequential Organ Failure Assessment, *D* the day after the diagnosis of severe sepsis, *GCC* glucocorticoid

When patients were divided into two groups according to their survival status on D28, there were no differences in hormone levels of the patients between two groups. Only APACHE II and SOFA scores were found to be significanly higher in patients who died.

Data of patients with SS were compared with 16 healthy people. Baseline STC, baseline and stimulated SaC levels were significantly higher in patients with SS with high STC than in healthy subjects. Baseline and stimulated cFC levels were found to be higher in high STC group than in low STC SS groups. But baseline and stimulated cFC levels were found to be lower in patients with high STC on D1 than control group. On D7, only baseline cFC levels were found to be higher in patients with high STC than in patients with low STC. Baseline and stimulated STC and cFC levels were found to be significantly lower in patients with low baseline STC than in healthy people, but SaC levels were found to be similar in two groups (Table 1). Serum CBG and albumin levels were found to be significantly lower in both SS groups than in healthy group (Table 1).

**Fig. 2:**
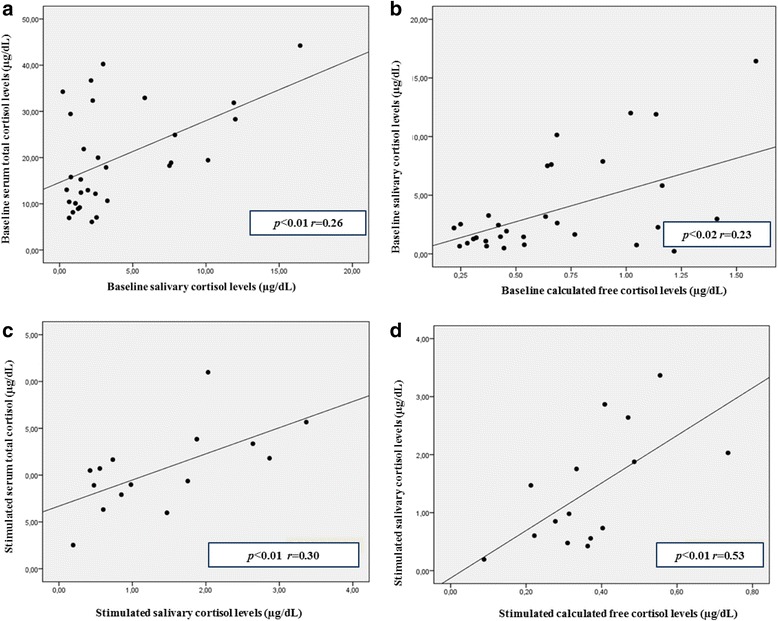
Correlations between STC, SaC and cFC in baseline and stimulated conditions

All simultaneous baseline SaC and STC levels of patients with SS were pooled, and 145 pairs were obtained. Similarly, all simultaneous SaC and STC responses of patients to low-dose ACTH stimulation test were also pooled, and 48 pairs were obtained. SaC and STC were positively correlated with each other (*p* < 0.01, *r* = 0.26 and *p* < 0.01, *r* = 0.30, respectively, for random and stimulated levels). SaC and cFC levels were also positively correlated with each other (*p* = 0.02, *r* = 0.23 and *p* < 0.01, *r* = 0.53, respectively, for random and stimulated levels) (Fig. 2).

## Discussion

In this study, baseline STC levels were found to be higher in high STC SS patients (suspected to have intact HPA axis) than in healthy subjects. This finding was supported by previous data [25, 26]. Not only baseline STC but also peak STC, baseline and peak SaC responses after ACTH stimulation were found to be significantly higher in high STC group than in both low STC and control group. So, the cortisol production is increased in order to cope with the severity of sepsis.

It is known that an appropriate response of HPA axis to CI is crucial for survival; so, both very high or low responses have been associated with increased mortality [27, 28]. In the present study, death was found to be associated with high cortisol levels. Many studies have demonstrated increased STC levels in sepsis, and the degree of the elevation is related to the severity of the illness [25–29]. Similarly, in a study by Yildiz et al., the mean baseline plasma cortisol concentration was significantly lower in survivors compared with non-survivors in sepsis [25]. In another, although baseline and stimulated STC levels were higher in sepsis group than their recovery status on the 14th day of sepsis, baseline and stimulated STC levels were not found to be related to mortality [29]. But in the present study, baseline and peak STC on D1 and baseline STC levels between D1–D7 tended to be lower in patients who survived. Both baseline and stimulated STC levels were lower in the present study than in the study of Yildiz et al. [29]. In their study, higher peak STC levels may be explained by the use of higher dose of synthetic ACTH for test, but baseline STC levels were also found to be higher. Patients group which enrolled the study by Yildiz et al. included not only patients with SS but also sepsis [29]. So, their study population was not homogenous. Discordance between the two studies may be explained by this scenario.

In the present study, serum CBG concentrations were found to be lower in SS than in healthy individuals compatible with prior studies [13, 30]. The lower CBG levels in sepsis can be explained by multifactorial causes such as decreased liver synthesis of CBG, proteolytic cleavage by neutrophil elastase, and less common genetic mutations [12, 30, 31]. And we have also shown that the CBG levels did not show an increase after ACTH stimulation.

As expected, stimulated STC levels were found to be lower in patients with SS who had low baseline STC levels than in healthy group. cFC formula strongly depends on STC, so similar tendency with STC and cFC levels is not surprising. One of the major limitations of the present study is that we have determined cFC levels by using STC and CBG measurements. So, cFC strongly reflects variations on STC and CBG levels. This explains the lower cFC levels obtained in SS subgroups than in the controls. If we could directly measure FC levels in patients with SS, we could do more certain judgement whether SaC levels better reflect or not serum FC levels.

Higher APACHE II and SOFA scores reflect worse clinical outcome. In many studies, SOFA or both SOFA and APACHE II scores were found to be related to mortality [9, 25, 29]. In the present study, the patients who did not survive also had higher APACHE II and SOFA scores. Although APACHE II and SOFA scores were not affected by the presence of low or high baseline STC levels, they were found to be related to mortality.

There was a significant correlation between SaC and STC and SaC and cFC. The relationship between SaC and STC is nonlinear; after a rapid increase in STC first, the serum CBG becomes saturated. CBG is saturated at a STC of 16 to 18 μg/dL. After STC exceeds CBG saturation, the free portion of cortisol increases [32]. It could explain why SaC and cFC levels do not increase simultaneously.

## Conclusions

In conclusion, SS is associated with increased SaC, but decreased cFC levels when baseline STC is assumed to be sufficient. When STC level is assumed to be insufficient, SaC levels remain unchanged, but cFC levels are decreased. Lower STC levels is not associated with increased mortality in patients with SS. Although STC levels are correlated with SaC and cFC levels in patients with SS, these findings should be validated in an independent cohort before considering SaC or cFC instead of STC for diagnosing AI in patients with SS.

## Abbreviations

ACCP: American College of Chest Physicians
ACTH: adrenocorticotrophic hormone
AI: adrenal insufficiency
APACHE II: Acute Physiology and Chronic Health Evaluation II
CBG: cortisol binding globulin
cFC: calculated free cortisol
CI: critical illness
CIRCI: critical illness related adrenal insufficiency
COPD: chronic obstructive pulmonary disease
D1: first day of diagnosis of severe sepsis
D28: 28th day of diagnosis of severe sepsis
D7: 7th day of diagnosis of severe sepsis
DM: diabetes mellitus
EI: Enzyme immunassay
FC: free cortisol
HPA: Hypothalamus-pituitary-adrenal
ICU: Internal Medicine Intensive Care Unit
RAI: relative adrenal insufficiency
SaC: salivary cortisol
SCCM: Society of Critical Care Medicine
SOFA: Sequential Organ Failure Assessment
SPSS: Statistical Package for Social Sciences
SS: severe sepsis
STC: serum total cortisol
